# *Brachypodium distachyon* Seedlings Display Accession-Specific Morphological and Transcriptomic Responses to the Microgravity Environment of the International Space Station

**DOI:** 10.3390/life13030626

**Published:** 2023-02-23

**Authors:** Shih-Heng Su, Howard G. Levine, Patrick H. Masson

**Affiliations:** 1 Laboratory of Genetics, University of Wisconsin-Madison, 425 G Henry Mall, Madison, WI 53706, USA; 2 NASA John F. Kennedy Space Center, Kennedy Space Center, Merritt Island, FL 32899, USA

**Keywords:** *Brachypodium distachyon*, Bd21, Bd21-3, Gaz8, microgravity, International Space Station, APEX Growth Unit, VEGGIE, adaptation, seedling growth, transcriptomics

## Abstract

Plants have been recognized as key components of bioregenerative life support systems for space exploration, and many experiments have been carried out to evaluate their adaptability to spaceflight. Unfortunately, few of these experiments have involved monocot plants, which constitute most of the crops used on Earth as sources of food, feed, and fiber. To better understand the ability of monocot plants to adapt to spaceflight, we germinated and grew *Brachypodium distachyon* seedlings of the Bd21, Bd21-3, and Gaz8 accessions in a customized growth unit on the International Space Station, along with 1-g ground controls. At the end of a 4-day growth period, seedling organ’s growth and morphologies were quantified, and root and shoot transcriptomic profiles were investigated using RNA-seq. The roots of all three accessions grew more slowly and displayed longer root hairs under microgravity conditions relative to ground control. On the other hand, the shoots of Bd21-3 and Gaz-8 grew at similar rates between conditions, whereas those of Bd21 grew more slowly under microgravity. The three Brachypodium accessions displayed dramatically different transcriptomic responses to microgravity relative to ground controls, with the largest numbers of differentially expressed genes (DEGs) found in Gaz8 (4527), followed by Bd21 (1353) and Bd21-3 (570). Only 47 and six DEGs were shared between accessions for shoots and roots, respectively, including DEGs encoding wall-associated proteins and photosynthesis-related DEGs. Furthermore, DEGs associated with the “Oxidative Stress Response” GO group were up-regulated in the shoots and down-regulated in the roots of Bd21 and Gaz8, indicating that Brachypodium roots and shoots deploy distinct biological strategies to adapt to the microgravity environment. A comparative analysis of the Brachypodium oxidative-stress response DEGs with the Arabidopsis ROS wheel suggests a connection between retrograde signaling, light response, and decreased expression of photosynthesis-related genes in microgravity-exposed shoots. In Gaz8, DEGs were also found to preferentially associate with the “Plant Hormonal Signaling” and “MAP Kinase Signaling” KEGG pathways. Overall, these data indicate that *Brachypodium distachyon* seedlings exposed to the microgravity environment of ISS display accession- and organ-specific responses that involve oxidative stress response, wall remodeling, photosynthesis inhibition, expression regulation, ribosome biogenesis, and post-translational modifications. The general characteristics of these responses are similar to those displayed by microgravity-exposed *Arabidopsis thaliana* seedlings. However, organ- and accession-specific components of the response dramatically differ both within and between species. These results suggest a need to directly evaluate candidate-crop responses to microgravity to better understand their specific adaptability to this novel environment and develop cultivation strategies allowing them to strive during spaceflight.

## 1. Introduction

On Earth, plants are exposed to a unidirectional 1-g force that guides the growth of their organs (gravitropism). Furthermore, the weight imposed by gravity on each organ results in a mechanical load that must be counteracted by thick, sturdy cell walls and specialized morphologies. Millions of years of evolution on Earth have assured the effectiveness of these gravitropic, gravi-resistance, and gravimorphogenesis processes, allowing plants to grow and develop properly, forming morphologies that effectively permit completion of their life cycle [1]. Spaceflight, on the other hand, imposes very different constraints on the plants, which they never encountered during their evolution on Earth.

During spaceflight, the directional cue provided by gravity is insufficient to modulate gravitropism, gravi-resistance, and other directional cues within the environment. Therefore other tropisms have to take over and guide plant organ growth, including light, gradients in water, oxygen, temperature, ions, and chemicals [2,3]. Similarly, the weightlessness associated with microgravity exposure implies a lack of weight-bearing on plant organs, leading to different mechanical constraints on their growth behaviors [1,4]. Other critical alterations of the physical environment encountered by plants during spaceflight include a lack of gravity-induced convection resulting in diminished gas exchange at the surface of the plant, altered photosynthesis, respiration, hypoxia, and thermoregulation [1,2,5,6,7,8]. Furthermore, exposure to elevated cosmic radiation can substantially impact the morphology, biology, and genetic makeup of plants during spaceflight [9].

Overall, the environmental cues that plants experience during spaceflight trigger a range of biological stress responses that lead to phenotypes collectively named “space syndrome”. These include shorter organs, thinner leaves, roots tending to grow away from the light source (negative phototropism) while also skewing in some cases, root hairs of altered size, modified organelle size and shape, altered metabolism, and accumulation of starch, to cite only a few [10,11,12,13,14].

Researchers have heavily relied on transcriptomic and proteomic profiling approaches to reveal key molecular pathways that are responsive to the spaceflight environment relative to ground controls. Experiments using mostly *Arabidopsis thaliana* seedlings or plant tissue cultures as experimental materials have revealed potential contributions of Ca^2+^, light and reactive oxygen species (ROS) signaling, cell wall remodeling, and defense responses in plant adaptation to the spaceflight environment [6,8,10,12,14,15,16,17,18,19,20,21,22,23]. Some of these expression changes were accompanied by alterations in the pattern of CNG and CNN DNA methylation in the proximity of the corresponding genes, suggesting some epigenetic contribution to the process [24]. Interestingly, a short root-hair phenotype displayed by Arabidopsis seedlings grown in the BRIC hardware onboard the Space Shuttle Discovery (STS-131) was accompanied by decreased expression of genes associated with oxidative stress (such as peroxidases) and cell wall remodeling, and reverse genetics demonstrated a contribution of some of these genes in modulation of root hair growth [14].

The profiling investigations described above have revealed a large variability in expression responses between experiments due in part to differences in hardware used to grow the plants, growth conditions, and plant genotypes (reviewed in [25]). The latter parameter (plant genotype) is particularly interesting to consider. Indeed, the geographical origin of a specific plant accession plays a key role in defining the type of responses the plant will develop under specific environmental conditions, reflecting an important role for selective forces in dictating the evolution of adaptive traits. Consequently, for any plant species, individual ecotypes carry specific sets of alleles that allowed the plants to not only survive and adapt to the conditions of their native environment, but also result in distinct abilities to cope with new environments to which they might subsequently become exposed. Therefore, it is not surprising that experiments attempting to evaluate Arabidopsis’s ability to adapt to the microgravity environment have revealed dramatic accession-specific differences in their ability to grow, develop, and respond molecularly to this novel environment.

In fact, most experiments aimed at investigating Arabidopsis transcriptional responses to spaceflight have been carried out using the Col ecotype at the seedling stage [10,14,15,20,26] or as cell cultures [18,21]. Additionally, transcriptomic responses to spaceflight of Ler-0 and Ws ecotypes have been investigated in multiple space environments such as in Space Shuttles [8] and the International Space Station (ISS) [17,22], using a variety of hardware such as the Advanced Biological Research System (ABRS: [10,15]) or the CARA experiment [16], respectively. More recently, a landmark paper described a more thorough comparative transcriptomic analysis of four Arabidopsis ecotypes (Col-0, Ws-2, Ler-0, and Cvi-0) exposed to either microgravity conditions or 1-g [6]. Altogether, these experiments demonstrated that different accessions display both common and accession-specific responses to the microgravity environment relative to ground controls. Shared transcriptomic responses included the activation of expression of molecular chaperones that may protect the cells against oxidative damage imposed by the hypoxic conditions that typically develop around plant organs in the absence of convective buoyancy under microgravity [6].

While *Arabidopsis thaliana* serves as an outstanding genetic model for investigations of dicot plant responses to the microgravity environment encountered during spaceflight, most major agriculture crops grown on Earth for food, feed, and fiber are monocots, which diverged from the dicots ~150 million years ago [27]. Unfortunately, comparatively fewer experiments have been carried out to investigate the effects of spaceflight on monocot plant growth, morphology, and expression profiles. The wheat PESTO experiment investigated the growth of semi-dwarf wheat plants (*Triticum aestivum* L. cv. USU Apo) onboard the ISS relative to 1-g ground controls, demonstrating the existence of morphological differences between these two groups, including thinner leaves with ovoid chloroplasts and greater packaging density for microgravity-exposed plants relative to ground controls [13]. Similar phenotypes were also displayed by microgravity-exposed *Zea mays* plants [28]. On the other hand, rice seedlings (*Oryza sativa* L. cv. Koshihikari) grown for 68.5–136 h in the Space Shuttle STS-95 mission displayed longer roots than ground control, a phenotype associated with some thinning of the cell wall along with higher elasticity modulus and viscosity coefficient in space relative to ground controls [29].

Shagimardanova and colleagues analyzed the effects of microgravity on the transcriptome of barley (*Hordeum vulgare*) plants growing in the Lada root module onboard the ISS [30]. They observed substantial transcriptomic differences between spaceflight and ground-control seedlings, including genes preferentially associated with stress response, pathogenesis, and antioxidant pathways [30]. On the other hand, rice seedlings (*Oryza sativa* L.) germinated and grown in a *Science in Microgravity Box* (SIMBOX) biological incubator on board the Shenzhou-8 spacecraft displayed dramatically altered photosynthesis characteristics with decreased photosystem 1 (PS1) efficiency relative to ground controls. This phenotype was associated with decreased abundance of proteins associated with the PS1, NAD(P)H dehydrogenase (NDH), and cytochrome b6f (Cytb6f) complexes. Hence, spaceflight may lead to declines in PSI activity in rice, either as a direct consequence of microgravity, or caused by side effects of spaceflight associated with inefficient airflow, high CO_2_ and ethylene levels, radiation, and/or other experimental parameters [31].

In this paper, we present the results of an experiment named APEX-06 (Advanced Plant Experiment-06) aimed at investigating the effect of spaceflight on the growth, morphology, and transcriptome profiles of *Brachypodium distachyon*, a genetic model for monocots closely related to cereal crops such as oat, wheat, barley, and rice [32]. We show that Brachypodium seedlings grown on the surface of foam substrates within modified Magenta-Box containers display accession-specific alterations in seedling organ growth onboard the ISS relative to ground controls. Surprisingly, exposure of Brachypodium to spaceflight conditions also triggered increased root-hair growth under our cultivation conditions, a phenotype that correlated with altered expression of key genes associated with ROS signaling and root-hair growth in one of the tested accessions. We also report dramatic differences in organ-specific transcriptomic responses to the spaceflight environment between Brachypodium accessions and discuss these observations within the context of monocot plant adaptation to spaceflight. Because a 1× *g* centrifugation control was not available on ISS for the VEGGIE growth chamber utilized in these experiments, the growth and transcriptomic responses to spaceflight observed in this project may be the consequence of exposure to microgravity, radiation, or other spaceflight-associated parameters such as inefficient airflow and gas exchange, high ethylene levels, and/or other parameters.

## 2. Materials and Methods

### 2.1. A Novel APEX Growth Unit Allows Transportation of Pre-Planted Dry Brachypodium distachyon Seeds to ISS and Subsequent Activation of Germination

Experiments aimed at investigating the effects of microgravity environments on plant growth, development, and gene expression relative to ground controls should be conducted such that germination and growth occur under microgravity for the treated plants, avoiding any post-germination exposure to the 1-g conditions of Earth and/or hypergravity and vibration conditions typically experienced during takeoff and flight back to Earth. When using the dicot model plant *Arabidopsis thaliana*, seeds can be planted in agar-based media on the ground, then treated with far-red light followed by darkness to prevent germination during spaceflight [33]. Unfortunately, this far-red light pretreatment is not sufficient to block the germination of imbibed *Brachypodium distachyon* seeds before launch [34]. Therefore, we designed a novel foam-based growth system named the **APEX Growth Unit**, which can be planted with quiescent dry Brachypodium seeds on Earth, then transported to the ISS where the experiment can be activated by injecting growth medium into the foam.

**Figure 1:**
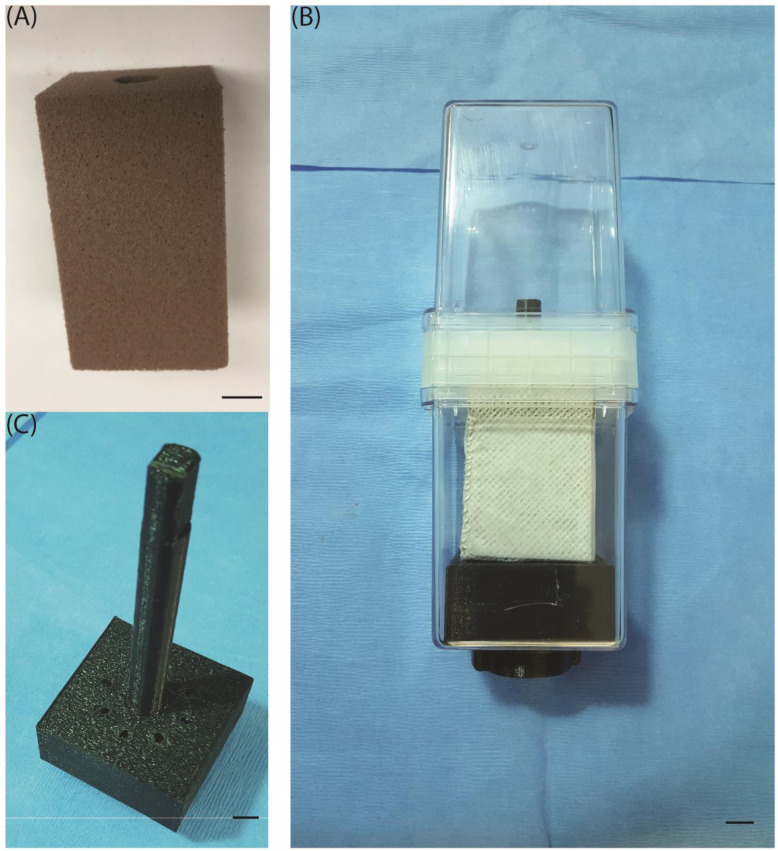
The APEX Growth Unit hardware. (**A**) The central Oasis Hortcube foam block (LC2, Oasis Grower Solutions) is used as a seed support during takeoff as well as following germination. It also serves as a nutrient reservoir during seedling growth. (**B**) The fully assembled APEX Growth Unit contains a foam block wrapped with a layer of medical gauze, allowing a more homogenous distribution of growth medium at the surface of the block where germinating seedlings are located. An outer layer of Nitex mesh prevents the roots of germinating seedlings from penetrating into the foam block, thereby hindering observation of their growth. (**C**) A growth-medium injection showerhead carries a central stick that serves as a support for a foam cube within the Magenta box. The injection showerhead is accessible for nutrient injection into the foam block through a port that crosses the Magenta box wall at its bottom. Black lines refer to 1 cm.

The APEX Growth Unit is made of a Magenta jar that contains a central Oasis Horticube foam whose general properties are described in the following link: https://oasisgrowersolutions.com/collections/horticubes%C2%AE (accessed on 6 February 2023) (Figure 1A) The foam block is wrapped with medical gauze and Nitex Mesch (Figure 1B) [35,36], and it is attached to an injection showerhead (Figure 1C). Surface-sterilized dry seeds are inserted in the foam before take-off, and germination is triggered by injection of sterile growth medium through the injection showerhead into the foam upon arrival on ISS. Plants growing on the surface of this foam block are contained within the Magenta jar, which maintains sterile conditions throughout the experiment. Several layers of medical gauze surrounding the nutrient-filled foam block assure better foam-to-root nutrient distribution during plant growth. Furthermore, a layer of Nitex mesh prevents roots from penetrating the foam block, assuring visibility of the root system throughout the experiment and facilitating root harvesting at the end of the growth period.

To elucidate the effect of microgravity on Brachypodium seedling growth and expression profile, we grew three *Brachypodium distachyon* accessions (Bd21, Bd21-3, and Gaz8) under both microgravity and ground-control conditions. For each accession, four biological repeats per condition were carried out, each including one APEX Growth Unit (described in Section 2) planted with 28 seeds on the four surfaces of the foam block. A first group of 12 APEX Growth Units was flown to ISS, where astronaut Scott Tingle initiated the experiment by injecting nutrient medium into the foam of each unit and positioning the units into an onboard VEGGIE growth chamber following the blueprint shown in Supplemental Figure S1. This arrangement was designed to minimize differential exposure between accessions to the light gradients existing between the center and corners of the VEGGIE growth unit. After the first day of exposure to red light to synchronize germination, seedlings were grown under red, blue, and green light for a total period of 5 days. At the end of the growth period, seedlings were photographed, harvested, and fixed in RNAlater for subsequent RNA-seq and RT-qPCR analysis.

A ground control was also carried out at Kennedy Space Center (KSC) with a two-day delay. This ground control mimicked the light, temperature, and CO_2_ conditions of the ISS experiment as well as the positions of the samples in VEGGIE. At the end of the growth period, ground control samples were photographed, harvested, and fixed in RNAlater following similar protocols as on ISS.

### 2.2. Plant Materials and Growth Conditions

Seeds of three *Brachypodium distachyon* accessions (Bd21, Bd21-3, and Gaz8) were obtained from Drs. Daniel Wood and Richard Amasino (University of Wisconsin–Madison). They were germinated and grown in soil (Berger Soil, BM2: Perlite: Vermiculite = 2:1:1) side-by-side in the same growth chamber exposed to a constant temperature of 22 °C, 65% relative humidity, 20 h day/4 h night cycles and 100–120 µmol/m^2^/s cool-white fluorescent light during the days. The plants were fertilized every other week with Blossom Booster Fertilizer (10-30-20). They were allowed to self-fertilize and generate seeds under these conditions. After harvesting, seeds were allowed to rest at room temperature for a minimum of three weeks to lift dormancy. At the time of the experiment, seeds were dehusked, surface-sterilized with five successive 1-min washes with 95% (*w*/*v*) ethanol, and then air-dried in a sterile hood for at least 20 min. Then, 24 seeds per APEX Growth Unit were inserted into the foam (6 per side), oriented with the embryo directed outward on the surface. Each accession was represented by a total of four APEX Growth Units (total of 96 seeds per accession). The seeded APEX Growth Units were packed in Cold Bags [37] held at 4 °C and transferred to the Dragon capsule on SpaceX-14 where they were stored for 3 days before launch.

### 2.3. ISS Experiment

SpaceX-14 launched on 2 April 2018, and docked with the ISS ~2 days later. Twelve APEX Growth Units (4 per accession) were unpacked by astronaut Scott Tingle on 12 April 2018, and the experiment was activated by injection of 90 mL half-strength Murashige and Skoog (MS: Sigma M5624 and MES: M2933) liquid medium into each APEX Growth Unit through the injection port (Figure 1). The APEX Growth Units were then integrated into the onboard VEGGIE (Vegetable Production System) growth unit fitted with Magenta-box holders following the pattern illustrated in Supplemental Figure S1 [38]. The red-LED lights were switched on to promote synchronized germination (low setting). After 24 h, the green and blue LED lights were also switched on (low setting) and the seedlings were allowed to grow for a period of 4 days under continuous light (blue, green, and red LEDs at an intensity of approximately 40–55 µmol/m^2^/s). HOBO environmental sensors/data loggers were placed into VEGGIE near the APEX Growth Units to capture the environmental conditions (temperature, humidity) during the entire growth period.

At the end of this growth period, seedlings were photographed with a Nikon D5 camera and then harvested into Kennedy Fixation Tubes (KFTs) preloaded with RNAlater (one KFT per APEX Growth Unit). Tissue fixation was initiated by forcing RNAlater from the storage chamber of the KFTs into the fixation chamber containing harvested plant tissue. Fixation proceeded at room temperature for a period of 24 h. The tubes were then stored in the Minus-Eighty Laboratory Freezer on the ISS (MELFI) for 20 days before being loaded onto the Dragon capsule and returned to Earth on 5 May 2018. The fixed samples were de-integrated from the KFTs and transferred into 50 mL Falcon Tubes at KSC, then delivered frozen at −80 °C to the Principal Investigator’s laboratory at the University of Wisconsin–Madison for molecular analysis.

### 2.4. Ground Control

A duplicated set of APEX Growth Units planted with seeds from the same three accessions was generated and used for a ground control experiment that was carried out in a VEGGIE growth chamber within the ISS Environmental Simulator (ISSES) at Kennedy Space Center (KSC). The ISSES was programmed to simulate the environmental conditions recorded by the HOBO sensors in the VEGGIE unit of the ISS, with a 2-day delay. The four APEX Growth Units associated with each of the three accessions were positioned within VEGGIE along a pattern that was identical to the one used in the ISS experiment (Supplemental Figure S1). The experimental protocol for this ground control experiment was identical to the one followed on the ISS.

At the end of the growth period, the seedlings were also photographed with a Nikon D4s camera, then harvested in KFTs and fixed in RNAlater as described for the ISS experiment. After 1-day fixation at room temperature, the samples were transferred to a −80 °C freezer, then sent to the Principle Investigator’s laboratory at the University of Wisconsin-Madison under dry ice for subsequent RNA extraction and expression analysis.

### 2.5. RNA Extraction and RNAseq Analysis

Frozen fixed seedlings were thawed, then dissected to separate shoots and roots. Total RNA was extracted from the root or shoot tissues using Direct-zol RNA Miniprep Plus extraction kits (Zymo Research, Irvine, CA, USA). DNAse-I treatment was performed in column using RNase-Free DNase-I Set (Qiagen, Valencia, CA, USA). RNA quality was monitored using Agilent 2100 Bioanalyzer and Eukaryotic total RNA NanoChip technologies (Agilent Technologies, Santa Clara, CA, USA). Approximately 1 µg total RNA from each sample was used to generate cDNA libraries with rRNA reduction using TruSeq Stranded Total RNAlibrary Prep Plant Kits (lllumina, San Diego, CA, USA). Paired-end sequencing (2 × 150 bp) was performed at the University of Wisconsin–Madison Biotech Center DNA Sequencing Facility using NovaSeq 6000 (Illumina, San Diego, CA, USA). Libraries were multiplexed with a target of ~60 million reads per sample for Bd21. For the other two accessions, Bd21-3 and Gaz8, RNA sequencing was performed by the National Aeronautics and Space Administration (NASA)’s GeneLab Sample Processing Lab (https://genelab.nasa.gov (accessed on 6 February 2023)) on a Illumina NovaSeq6000. Paired-end sequencing (2 × 100 bp) reactions were performed with ~120 million reads per sample. The corresponding sequencing fastq files have been uploaded into the GeneLab Data Repository (https://osdr.nasa.gov/bio/repo/data/studies/OSD-375 (accessed on 6 February 2023); OSD-375; GLDS-375).

### 2.6. RNA Sequence Mapping and Transcriptional Profiling

The Tuxedo pipeline was used to map RNAseq sequence reads to the *Brachypodium distachyon* accession Bd21 reference genome version 3.0 (Phytozome) [39]. Briefly, the paired-end 150-bp (Bd21) or 100-bp (Bd21-3 and Gaz8) sequence reads were joined after filtering out low-quality bases with Phred scores below 30. TopHat2 was then used to map the splice junctions between exons after aligning the reads to the *Brachypodium distachyon* accession Bd21 reference genome version 3.0 (Phytozome) using Bowtie2 as alignment engine; 2-bp mismatches were allowed in this step. The percentages of sequence reads aligned to the reference genome for each sample are summarized in Supplemental Table S1. The overall read mapping rate averaged 75% to 80%, except for one of the Gaz8 root samples in the ground control (B3 position), which displayed a mapping rate of 41.7%. Considering the lower mapping rate for this sample, we excluded it, along with the matched B3 Gaz8 flight sample, from subsequent analyses. HTseq was used to calculate the number of reads assigned to each annotated transcript. The DEseq [40] and EdgeR [41] R-based packages were used to normalize the data and evaluate differential expression between microgravity-exposed and ground control samples. Four biological replicates per accession were used to identify differentially expressed genes. *p*-values were adjusted for multiple testing using either the Benjamin–Hochberg method (for DEseq) or the q-values set to false discovery rates (FDR) of 0.05 (for EdgeR). Thresholds for significant differences were set at *p*(*q*) < 0.05.

### 2.7. Bioinformatic Analysis, Annotation, and GO Enrichment of Differentially Expressed Genes

Differentially expressed genes (DEGs) were annotated using the information available in Phytozome for *Brachypodium distachyon* accession Bd21 (*Brachypodium distachyon Bd21* database, version 3.1; https://phytozome-next.jgi.doe.gov/info/Bdistachyon_v3_1 (accessed on 6 February 2023)) [42]. Gene ontology enrichment analysis used the agriGO platform (http://systemsbiology.cau.edu.cn/agriGOv2/ (accessed on 6 February 2023)). Singular enrichment analysis (SEA) was chosen for all enrichment analyses. Gene identification numbers (*Bradi*) associated with each annotated gene in Phytozome were directly used as input for all three accessions. For reactive oxygen species (ROS)-associated DEGs, assignment to defined ROS-wheel categories was performed as defined in [43], using Arabidopsis orthologs to the Brachypodium DEGs as input. Predicted Arabidopsis orthologs to Brachypodium DEGs were obtained using Phytozome database (https://phytozome-next.jgi.doe.gov/info/Bdistachyon_v3_1 (accessed on 6 February 2023)). The threshold for significant enrichment was set at FDR = 0.05 [44,45]. For protein–protein interaction analysis, the STRING platform was used (https://string-db.org/ (accessed on 6 February 2023)) to identify potentially interacting DEG partners. GO enrichment and KEGG predictions were obtained directly from the STRING output [46].

For cis-acting element identification, we obtained the genomic sequence of the DEG’s transcribed region along with 5 kb of upstream and downstream sequences and used it as input data in the PLACE database to identify possible conserved cis-acting elements in proximity of each DEG (https://www.dna.affrc.go.jp/PLACE (accessed on 6 February 2023)) [47].

For transcription factor prediction and transcriptional regulation network analysis, Bd21 and Gaz8 shoot down-regulated DEGs belonging to the Photosynthesis GO group (GO:0015979) were identified and their Arabidopsis orthologs characterized. PlantRegMap (http://plantregmap.gao-lab.org/index.php (accessed on 6 February 2023)) was then used to search for possible transcription factors (TFs) regulating these DEGs [48]. This list of candidate TFs was compared to the full list of DEGs, allowing the identification of TFs that are differentially expressed in microgravity conditions relative to ground controls. These lists allowed us to reconstruct possible regulatory networks, which were manually drawn using Adobe Illustrator.

### 2.8. Morphology Measurement

High-resolution seedling photographs (with scale) were taken on the ISS and in ground control experiments at the end of the growth period before tissue harvesting and fixation. Shoot and root lengths were measured separately using ImageJ (https://imagej.nih.gov/ij/ (accessed on 6 February 2023)). All germinated seedlings were measured for each accession (*n* > 68). For root-hair length, the resolution of the photographs was not sufficient to allow determination of hair sizes along each photographed root. Therefore, root-hair length was quantified on only a portion of the seedlings analyzed. For each image, all roots that did not overlap with other roots and whose root hairs were clearly defined and measurable were used. Root-hair length was measured in a region of the root located approximately 3 cm above the root tip, within the mature zone, where the root hairs have reached full-length (*n* > 10 roots for each accession and growth condition). Student’s *t*-test with 2-tailed distributions and equal variances was used to determine the statistical significance of observed differences (*p* < 0.05), using Microsoft Excel.

### 2.9. qRT-PCR Analysis

1 μg total mRNA per sample was reverse-transcribed into cDNA using the qScript cDNA SuperMix and following a protocol recommended by the supplier (QuantaBio, Beverly, MA, USA). Then, 250 ng RNA-equivalent cDNA was used to perform RT-qPCR per reaction. The RT-qPCR reactions (250 ng cDNA, 0.2 μM primers, and 1× Bullseye EvaGreen qPCR Mastermix; MidSci, St. Louis, MO, USA) were run on a LightCycler 480 II Instrument (Roche, Basel, Switzerland). Gene expression was calculated using the delta-Ct approach, and the data were standardized to the *UBC18* (*Bradi4g00660*) reference gene [49]. All flight expression levels were standardized to ground control levels. Student’s *t*-test with 2-tailed distributions and equal variances was used to determine the statistical significance of observed differences between samples (*p* < 0.05), using Microsoft Excel (version 2009).

## 3. Results

### 3.1. Microgravity Affects Seedling Growth in an Accession-Specific Manner

**Figure 2:**
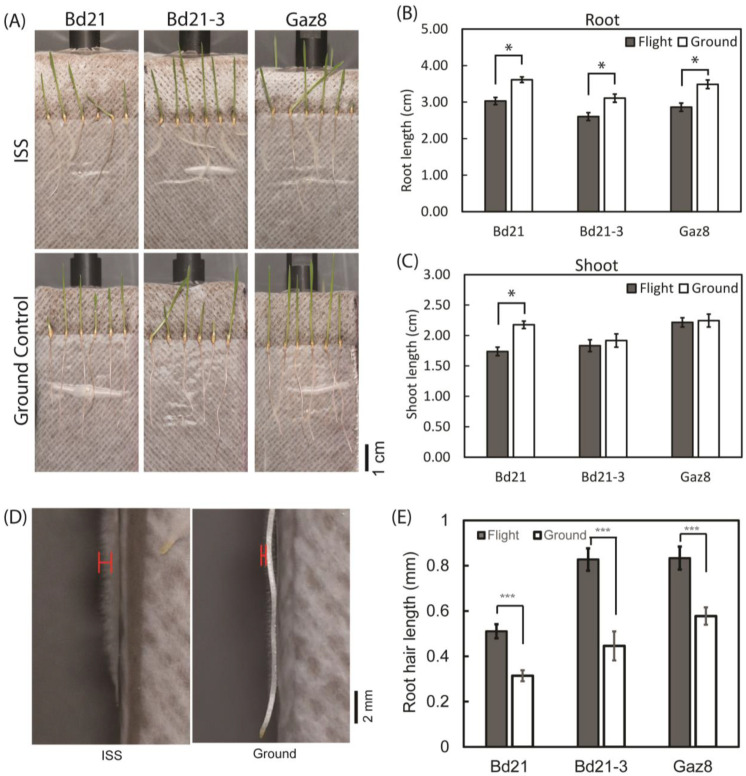
*Brachypodium distachyon* seedlings display accession-specific morphological alterations in the microgravity environment of ISS relative to ground controls. Accessions include Bd21, Bd21-3, and Gaz8. (**A**) Representative 5-day-old Brachypodium seedlings of the Bd21, Bd21-3, and Gaz8 accessions grown on the ISS (upper panel) and on Earth (lower) panel). Biometric analysis of (**B**) root length and (**C**) shoot length of 5-day-old *Brachypodium distachyon* seedlings from the Bd21 (*n* > 69), Bd21-3 (*n* > 68), and Gaz8 (*n* > 68) accessions. (**D**) Close-up view of representative roots of 5-day-old Gaz8 seedlings, showing differences in root-hair lengths between microgravity-exposed (**left**) and ground-control (**right**) seedlings. Representative root-hair length measurement is marked in the red line. (**E**) Quantitative measurement of root hair sizes between microgravity-grown and ground-control 5-day-old seedlings from the Bd21, Bd21-3, and Gaz8 accessions. Based on the resolution of the images, each root was measured once in the root mature zone from the root hair tip to the approximate root epidermal layer (*n* > 10). In **B**, **C**, and **E**, *t*-tests were used to determine the significance (* *p* < 0.05; *** *p* < 0.001; *n* > 10).

In a first attempt to evaluate the effect of the microgravity environment of ISS on Brachypodium seedlings, we investigated the direction of root and shoot growth after five days of cultivation on ISS and a ground control at KSC, and quantified shoot and root growth from pictures taken at the end of the growth period under both conditions. Seedlings grown under microgravity showed more variation in shoot and root growth direction than those grown on the ground (Figure 2A), as expected considering the absence of gravi-response under microgravity. However, seedling organs were not randomly oriented. Shoots generally grew toward the light canopy whereas roots tended to grow away from it (Figure 2A). These directional growth trends may be partly due to the original orientation of the seeds embedded into the foam. However, phototropism may also offer directional guidance under microgravity.

Morphologically, microgravity-exposed seedling roots were 16% to 18% shorter than ground controls for all three accessions tested at the end of the growth period (Figure 2B). For shoots, we only saw a 20% reduction of growth under microgravity for Bd21, whereas Bd21-3 and Gaz8 shoots grew similarly under both conditions (Figure 2C).

In addition to observing differences in root and shoot growth under microgravity relative to ground controls, we also noticed a clear root-hair phenotype associated with growth under microgravity conditions. Indeed, the roots of microgravity-grown Brachypodium seedlings of all accessions were hairier than those grown on earth, at least for areas of the roots in direct contact with the feeding-block surface (Figure 2D). Unfortunately, we could not determine if this hairy-root phenotype was associated with increased root-hair density under microgravity. However, representative images from seedlings of all accessions tested were of sufficient resolution to allow the measurement of root-hair sizes. In all cases, root hairs were significantly longer on microgravity-exposed seedlings than ground controls (Figure 2D,E). Overall, these results suggest the existence of genetic variation between Brachypodium accessions for seedling growth responses to microgravity.

### 3.2. Microgravity-Exposed Seedling Roots and Shoots Display Distinct Transcriptomic Profiles Compared to Ground Controls

To evaluate the impact of spaceflight on gene expression, we compared the transcriptome profiles between microgravity conditions (ISS) and ground controls for dissected Bd21, Bd21-3, and Gaz8 *Brachypodium distachyon* seedling roots and shoots using the DEseq and EdgeR statistical packages. Candidate genes identified by both packages as displaying significant differences in expression between microgravity and ground control conditions are discussed below.

**Figure 3:**
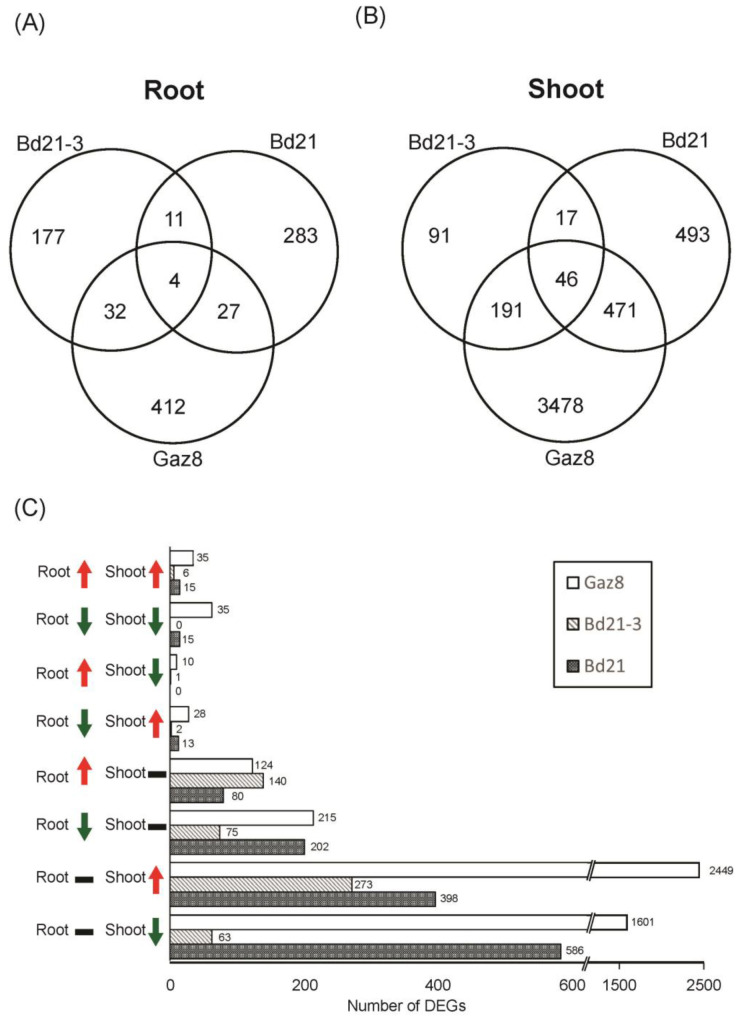
Transcriptome analysis of Brachypodium seedlings grown under the microgravity environment of ISS and ground control. (**A**,**B**) Venn diagram summarizing the numbers of genes showing differential expression in roots (**A**) and shoots (**B**) between microgravity conditions and ground control for the three Brachypodium accessions analyzed here (Bd21, Bd21-3, and Gaz8). Genes showing statistically significant differences between these two conditions using two statistical packages (DESeq and EdgeR) are reported here. (**C**) Numbers of genes showing significant increases (red upward arrows), decreases (green downward arrows), or no change (horizontal black bar) in expression between microgravity and ground-control conditions in shoots and roots of *Brachypodium distachyon* seedlings of the Bd21, Bd21-3, and Gaz8 accessions.

The three Brachypodium accessions tested in APEX-06 showed distinguishable expression responses to the microgravity environment of ISS. A total of 1027 and 325 genes were found to be differentially expressed (DEGs) between microgravity-exposed and ground-control conditions in shoots and roots, respectively, for Bd21, which is the accession traditionally used as a reference in Brachypodium genome sequencing projects (Figure 3A,B; Supplemental Table S2). Bd21-3, on the other hand, displayed fewer DEGs under microgravity conditions relative to ground controls, including 345 DEGs in shoots and 224 in roots (Supplemental Table S3). For Gaz8, 4186 and 475 genes were differentially expressed in shoots and roots (Supplemental Table S4), respectively (Figure 3A,B). This implies that different Brachypodium accessions may have distinct abilities to adapt to the microgravity environment of ISS based on the dramatic differences in expression responses to microgravity they displayed in this experiment.

Within each accession, very few DEGs were found to overlap between shoots and roots. In fact, a vast majority of DEGs were differentially expressed in only shoots or roots (Figure 3C). This observation held true for all accessions tested in this study.

When comparing accessions, only 46 and four DEGs were found to be shared by all three accessions in shoots and roots, respectively. We define these genes as common DEGs (Supplemental Table S5). The common DEGs share several interesting features. First, most of these common DEGs display expression changes in the same direction (up or down) under microgravity conditions relative to ground controls in all three accessions (with only one exception in the shoot samples). This observation may suggest that the common DEGs represent a significant component of plant responses to the microgravity conditions on ISS. Second, we performed a GO enrichment analysis of these common DEGs, using their Arabidopsis orthologues as queries. While the list of common root DEGs was too small to warrant such an analysis, we noticed an enrichment of the list of down-regulated common shoot DEGs for genes associated with the Photosynthesis (GO:0015979; FDR = 8.6 × 10^−9^), Photosynthesis/Light Reaction (GO:0019684; FDR = 7.4 × 10^−7^), and Generation of Precursor Metabolites and Energy (GO:0006091; FDR = 6.3 × 10^−5^) GO groups. All these GO groups belong to the Photosynthesis-Related hierarchy (Supplemental Table S5). This observation is consistent with previous studies in Arabidopsis [50] and rice [31], and also with our observation discussed in the following section that photosynthesis-related Gene Ontologies (GOs) are enriched in the general lists of down-regulated shoot DEGs in all three accessions (Supplemental Figure S4). Third, several common DEGs found in our list encode cell-wall-related proteins, including two up-regulated wall-associated kinases (WAKs) in shoots and one up-regulated laccase and one down-regulated peroxidase in roots (Supplemental Table S5). This is also consistent with previous studies in rice or Arabidopsis, indicating differential expression of genes associated with cell wall composition and/or extensibility under microgravity conditions [22,51,52,53].

### 3.3. GO Enrichment Analysis Suggests That Brachypodium Roots and Shoots Deploy Distinct Biological Strategies to Cope with the Microgravity Environment on ISS

**Figure 4:**
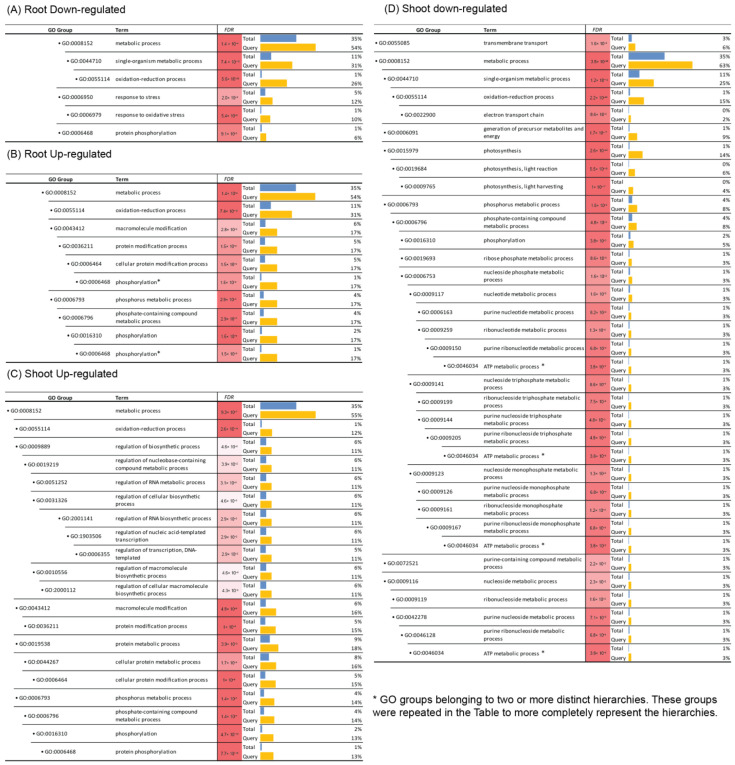
Gene Ontology (GO) enrichment analysis of DEGs from 5-day-old *Brachypodium distachyon* seedlings of the Bd21 accession exposed to microgravity conditions of ISS relative to ground-control conditions. All DEGs from Bd21 are split into four categories based on tissue (root/shoot) and expression regulation under microgravity relative to ground control (up/down): (**A**) root down-regulated DEGs, (**B**) root up-regulated DEGs, (**C**) shoot down-regulated DEGs, and (**D**) shoot up-regulated DEGs. Enrichment significance is represented by both FDR numbers and color shading, with darker red representing smaller FDR values. Blue bars represent expected values (total number of genes representing the GO group divided by the total number of genes in the genome), whereas yellow bars represent observed ratios (number of DEGs belonging to the GO group divided by the total number of DEGs). The GO groups are arranged along a hierarchical tree of biological processes. The original information was generated by using the agriGO website (http://bioinfo.cau.edu.cn/agriGO/ (accessed on 6 February 2023)). The singular enrichment analysis (SEA) method was used to calculate significance. * refers to the GO groups that belong to two or more different hierarchies and are repeated in the table to better represent the hierarchies.

To understand the biological processes that may be affected by the microgravity environment, we analyzed our list of DEGs for possible GO enrichments. First, we split the DEG list into four categories for each accession based on their up- or down-regulation in root and shoot tissues, respectively. Figure 4 shows the list of GO groups over-represented in Bd21. Among all four categories, DEGs are enriched for genes associated with the Oxidation-Reduction Process (GO: 0055114) (Figure 4). However, the DEGs belonging to this GO group were distinct between shoot and root tissues (Supplemental Table S6). In the root down-regulated category, most DEGs belong to the peroxidase and cytochrome P450 super families. On the other end, only a few peroxidases were identified in the shoot down-regulated category. Instead, this category contained more photosynthesis-related genes. Moreover, seven peroxidase genes were observed in shoot up-regulated DEGs (Supplemental Table S6), indicating opposite peroxidase expression trends between root and shoot tissues under the microgravity environment for Bd21.

Next, we expanded our GO enrichment analysis to DEGs identified in all three accessions analyzed in this project. Interestingly, the Oxidation-Reduction process (GO: 0055114) was found to be the only GO group enriched in all four categories of DEGs for all three accessions, reemphasizing the importance of the Oxidation-Reduction process in Brachypodium seedling responses to the microgravity environment on ISS (Supplemental Figures S2–S5).

**Figure 5:**
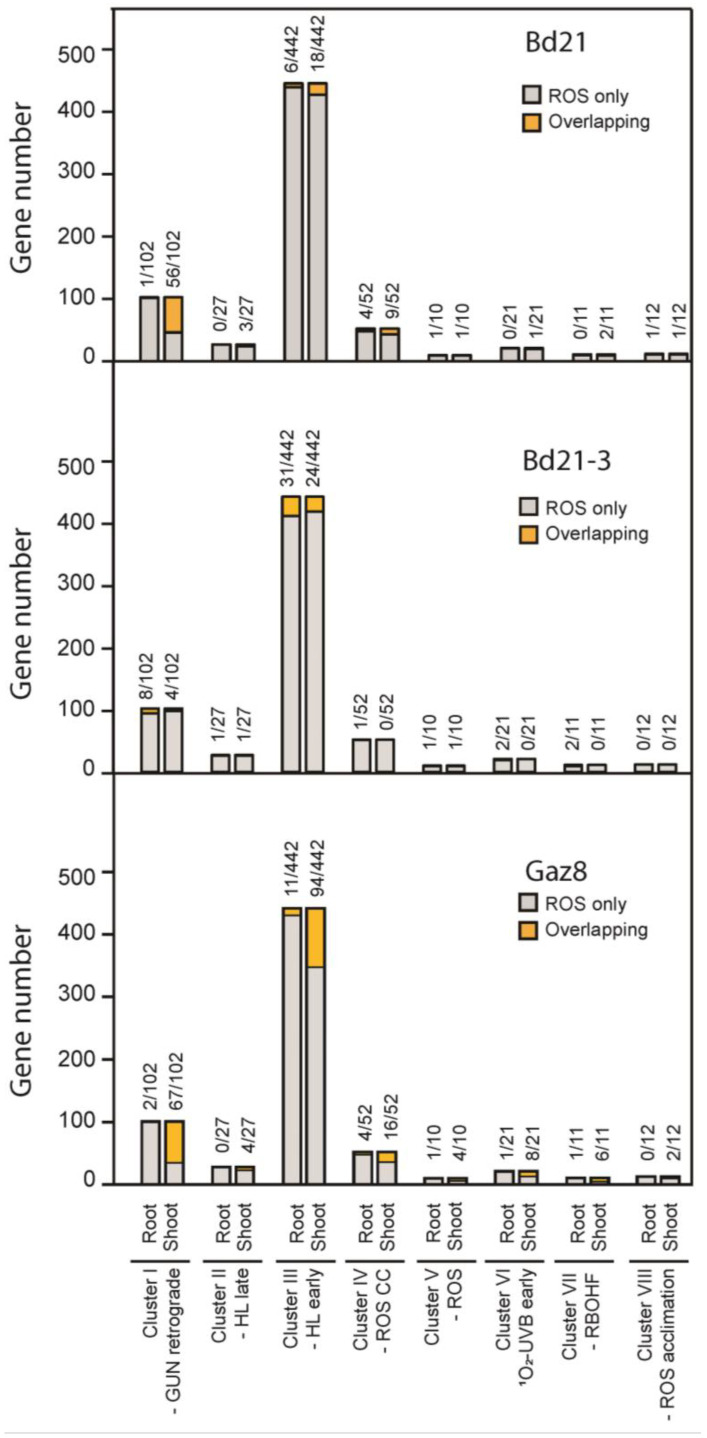
Arabidopsis orthologs to Brachypodium root and shoot DEGs with ROS-signaling annotation preferentially associate with specific transcriptional response clusters from the ROS wheel (Willems et al. (2016)). Clusters I-VIII refer to the categories of ROS-related experiments used to establish clusters of transcriptional responses in the ROS wheel meta-analysis. Bars represent the total number of genes present in each cluster. The proportions of genes present in a specific cluster that are also orthologous to specific microgravity-related Brachypodium DEGs (from this study) are represented in yellow.

Previous studies have shown that transcriptomic responses to a variety of redox homeostasis perturbation experiments in Arabidopsis cluster into eight main groups (the “ROS wheel”), each showing similar patterns of expression changes in response to distinct classes of perturbations [43]. Because our study revealed that the Oxidative-Reduction Process GO group is significantly enriched in the Brachypodium shoot and root transcriptomic responses to the microgravity environment on ISS, we compared our list of DEGs from Bd21 to the clusters identified in the ROS wheel. To do this, we used “best-hit annotation” (https://phytozome.jgi.doe.gov/pz/portal.html (accessed on 6 February 2023)) to identify Arabidopsis orthologs to our DEGs. From the 325 Bd21 root DEGs identified in our study, 296 have Arabidopsis orthologs, including 259 with unique orthologs. Further, 883 of the 1027 shoot DEGs were also found to have Arabidopsis orthologs, including 797 with unique orthologs (Supplemental Table S7 and S8). The lists of Arabidopsis orthologs to our DEGs were then compared to the lists of core genes associated with each cluster within the ROS wheel (Figure 5, Supplemental Table S9). Of the core genes identified in cluster I of the ROS wheel, 55% were orthologous to Bd21 shoot DEGs in our experiment. This result suggests that Brachypodium Bd21 shoots display expression responses to the microgravity environment on ISS that are similar to those displayed by Arabidopsis plants exposed to redox perturbations associated with defects in the GUN retrograde signaling pathway [54].

We performed a similar analysis in the Bd21-3 and Gaz8 accessions (Figure 5; Supplemental Table S9). Arabidopsis orthologs to Bd21-3 shoot DEGs associated with ROS signaling clustered preferentially with group-3 of the ROS wheel, representing early responses to high-light conditions, although this association did not reach statistical significance (chi-squared test; *p* = 0.54). For Gaz8, Arabidopsis orthologs to shoot DEGs preferentially clustered with both groups 1 and 3 of the ROS wheel (Figure 5; chi-squared test; *p* = 9.79 × 10^−11^). Together, these puzzling results suggest the existence of accession-specific responses to oxidative stress under microgravity conditions possibly triggered by differential organ sensitivities to distinct microgravity environment-related stimuli. Our results also suggest that Brachypodium seedlings grown under microgravity condition may use the retrograde signaling pathway to modulate their responses to the microgravity-related stressors and acclimate accordingly.

### 3.4. Brachypodium Genes Associated with Photosynthesis Are Down-Regulated under Microgravity Conditions on ISS

As mentioned in the previous sections, the oxidation-reduction process is important for Brachypodium seedlings growing under microgravity condition. When we compared the ROS wheel with our DEG lists, cluster I from the ROS wheel was significantly enriched within the Bd21 and Gaz8 shoot DEGs (Figure 5). Cluster I is enriched with photosynthesis-related genes [43]. Therefore, these results match our observation that Bd21 and Gaz8 shoot down-regulated DEGs are enriched with photosynthesis-related GO groups (GO.: 0015979, GO: 0019684, GO:0006091) (Supplemental Figure S4). This observation is also consistent with previous reports from Arabidopsis [50,55] and rice [31].

To understand the possible regulatory pathways leading to the down-regulation of photosynthesis-related Brachypodium genes under microgravity conditions, we sought transcription factors whose annotations predict a contribution to expression regulation of the photosynthesis-related DEGs in Bd21. Unfortunately, analyses using the Brachypodium and rice transcription-factor databases (http://plantregmap.gao-lab.org/index.php (accessed on 6 February 2023)) were not sufficiently robust to identify significant DEG expression regulators. Therefore, we used the Arabidopsis orthologs to our DEGs to search for possible regulatory networks.

**Figure 6:**
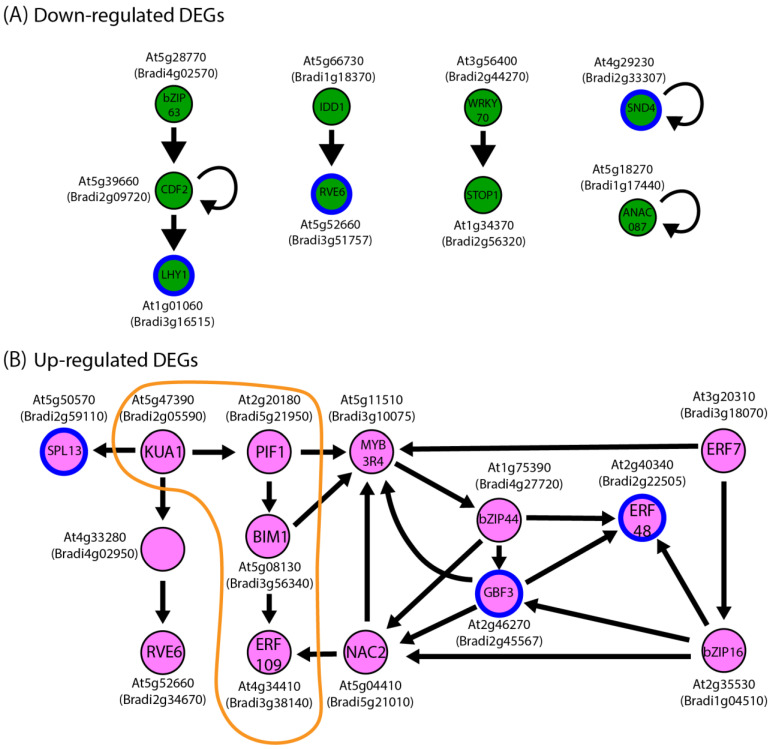
Arabidopsis transcriptional regulation networks involving transcription factors predicted to regulate DEG orthologs associated with the Photosynthesis GO group in Bd21. Orthologs to down-regulated (**A**) and up-regulated (**B**) transcription factors are shown in this figure. Nodes refer to the transcription factors and arrows indicate the direction of transcription regulation. Green represents down-regulation under microgravity whereas pink refers to up-regulation. Genes that are also orthologous to Gaz8 DEGs are surrounded by a blue circle with the same expression pattern. A regulatory pathway involving the *PIF1* gene is surrounded by a yellow outline. See Supplemental Figure S6 for a more detailed description of the *PIF1* regulatory network including target genes orthologous to Brachypodium DEGs.

In the Arabidopsis genome, a total of 252 genes are annotated as associated with the photosynthesis GO group (GO:0015979). Among them, 99 were orthologous to genes within our DEG list. We used these 99 DEG orthologs as input to retrieve 29 candidate transcription factors that are predicted to regulate these DEGs in Arabidopsis. Brachypodium orthologs to these candidate regulatory transcription factor genes were then identified and compared to our list of DEGs in the Bd21 accession (Supplemental Table S10). This analysis allowed us to build the predicted regulatory networks connecting transcription factor genes associated with photosynthesis-related Bd21-DEG orthologs in *Arabidopsis thaliana* (Figure 6). Several short linear pathways were identified amongst the dow-regulated DEG orthologs (Figure 6A). On the other end, a more complex, branched regulatory network could be built connecting numerous up-regulated DEG orthologs (Figure 6B). Within this up-regulated network, one pathway has been more thoroughly investigated in *Arabidopsis thaliana*, involving the *PHYTOCHROME-INTERACTING FACTOR1 (PIF1,* also named *PHYTOCHROME-INTERACTING FACTOR3-LIKE5; PIL5; At2g20180/Bradi5g21950*) (Supplemental Figure S6, surrounded by an orange line). This transcription factor preferentially interacts with the Pfr forms of Phytochrome A (PhyA) and Phytochrome B (PhyB) to modulate light effects in plants. Interestingly, the *Phytochrome A* (*PHYA; Bradi1g10520*) was also up-regulated in response to the microgravity environment in Brachypodium Bd21 shoots (Supplemental Table S2). Furthermore, up-regulation of *PIF1* was previously reported to inhibit photomorphogenesis-related processes in several plant species, including chlorophyll biosynthesis and photosynthesis in Arabidopsis [56,57,58,59].

To evaluate a possible effect of microgravity-induced activation of expression of the Brachypodium *PIF1*-ortholog on the expression of photosynthesis-related genes and processes in Bd21 shoots, we compared our list of Arabidopsis orthologs to the Bd21 shoot DEGs with a list of PIF1/4/5- (PIF-trio) and PIF3-regulated genes in Arabidopsis (obtained from Supplemental Tables S9 and S10 of Zhang et al., 2013 [60]). Supplemental Table S11 shows a total of 88 Brachypodium shoot DEGs (64 down-regulated and 24 up-regulated DEGs) whose Arabidopsis orthologs are either AtPIF1/4/5 (PIFtrio)- and/or AtPIF3-regulated genes, with similar patterns (directions) of expression changes. This list of putative PIF-regulated genes is strongly enriched for photosynthesis-related GO groups (GO: 0015979, GO:0006091, and GO:0009765…etc.), as expected (*p* ≤ 7.6 × 10^−29^).

To confirm the differential expression of these potential Brachypodium PIF-target genes under microgravity conditions relative to ground controls, we used a RT-qPCR approach to quantify the expression of both *PHYA (Bradi1g10520)* and *PIF1 (Bradi5g21950)* along with four randomly picked DEGs from the list of Brachypodium orthologs to Arabidopsis PIF-response genes (*Bradi3g07190, Bradi4g30060, Bradi2g16290, and Bradi1g1213*). Results shown in Supplemental Figure S7 demonstrate a significant up-regulation of both *PHYA* (*Bradi1g10520*; 5-fold) and *PIF1* (*Bradi5g21950*; 7-fold) in Bd21 under microgravity conditions relative to ground controls. It also demonstrates a down-regulation of all four DEGs under microgravity conditions relative to ground controls, in agreement with our RNAseq results.

Taken together, our results suggest that up-regulation of *PHYA* and *PIF*-gene expression under microgravity correlates with the down-regulation of photosynthesis-related genes in Brachypodium Bd21 shoots. Considering that Brachypodium is only distantly related to Arabidopsis, it will be important to directly test this hypothesis by evaluating the effects of *Bradi5g21950* activation on expression regulation and photosynthesis related processes in *Brachypodium distachyon*.

While our hypothesis of a role for *Bradi5g21950* (Brachypodium *PIF1* ortholog) and *Bradi1g10520 (PHYA)* in mediating the effect of microgravity on photosynthesis-related processes in Bd21 seems reasonable, the situation for Gaz8 may be more complex. Indeed, in Gaz8, seedling exposure to the microgravity environment on ISS appeared to either not affect or down-regulate the expression of *PHYA* and *PIF*-related genes (Supplemental Table S4). Furthermore, an analysis of photosynthesis-related Gaz8 DEGs identified 102 transcription factor genes as differentially expressed in Gaz8 under microgravity conditions. Yet, only six of these photosynthesis-related Gaz8 transcription factor DEGs were similar to those discussed above for Bd21 (Figure 6, blue circles). We conclude that another, yet unknown, mechanism may be responsible for regulating expression of photosynthesis-related genes in Gaz8. Whether such an alternative regulatory mechanism may also function, at least partially, in the regulation of photosynthesis-related genes in Bd21 and/or Bd21-3, remains unknown.

### 3.5. Genes Annotated as Potential Contributors to Transcriptional and Post-Translational Regulation Are Up-Regulated in Microgravity-Grown Bd21 and Gaz8 Shoots

In addition to the GO groups associated with plant responses to oxidative stress or photosynthesis discussed in the previous section, we noticed that two main hierarchies of GO groups associated with the Regulation of Transcription, DNA-Templated (GO:0006355) and Protein Modification (GO:0036211) are enriched in the microgravity up-regulated shoot DEGs in both Bd21 and Gaz8 accessions (Supplemental Figures S5). The DEGs associated with these two GO groups are listed in Supplemental Tables S12 and S13.

In Bd21, 23 DEGs are associated with the Regulation of Transcription, DNA-Templated (GO:0006355). Annotation of best-hit Arabidopsis and rice orthologs to these DEGs suggests that most of them encode transcription factors [42] (Supplemental Table S12). Four of them are predicted to encode ethylene response-element binding proteins or ERFs (Bradi2g27920, Bradi3g18070, Bradi3g38140, and Bradi2g22505), suggesting activation of the ethylene signaling pathway; another two of these transcription factors have been associated with ABA signaling (Bradi2g45000, and Bradi2g45567), potentially contributing to the plant space–stress mitigation.

Within the DEGs associated with the Protein Modification GO group (GO:0036211), the *Phytochrome A* (*Bradi1g10520*) gene discussed in the previous section may be an important modulator of plant responses to the microgravity environment of ISS. Additionally, 26 out of 31 DEGs associated with this GO group encode kinases. Of them, 15 are annotated as receptor-like protein kinases (RLKs), including one annotated as brassinosteroid-receptor kinase-like protein (BRL1) (Supplemental Table S13). It is worth noting that RLKs have been shown to contribute to plant responses to biotic and abiotic stressors as well as cell development [61,62,63]. Finally, this list of DEGs associated with GO:0036211 also includes several wall-associated protein kinase (WAKs) genes. Proteins belonging to this group straddle the plasma membrane, connecting the wall with the cytoplasm. Their extracellular domain has been shown to interact with both proteins and pectins in the cell wall whereas their cytoplasmic segment contains a kinase domain that transduces information derived from wall integrity, pathogen invasion, cell shape, deformation, and mechanostimulation, into cellular responses. Microgravity-induced expression of such wall associated protein kinase genes was also reported in *Arabidopsis thaliana* [64].

In Gaz8, 112 up-regulated shoot DEGs are associated with the Regulation of Transcription, DNA-Templated GO group (GO:0006355) and 147 are associated with the Protein Modification group (GO: 0036211) (Supplemental Tables S12 and S13). As in Bd21, multiple ethylene-responsive transcription factors (ERFs) are present in this list of shoot up-regulated DEGs. A couple of auxin response factor genes are also present in this list, as are several *WRKY* genes not found in the list of differentially expressed DEGs in accession Bd21 (Supplemental Table S12).

The list of Gaz8-up-regulated DEGs associated with Protein Modification (GO:0036211) includes a majority of genes annotated as kinases, including three that encode kinases associated with the brassinosteroid signaling pathway and several WAKs, as also observed in Bd21. Additionally, this list also includes a large group of genes that encode calcium/calmodulin-dependent protein kinases, which are not differentially expressed in Bd21. This is noteworthy because calcium and calmodulin have been suggested to play important roles in gravity signal transduction and mechanotransduction in Arabidopsis [65]. Finally, the list of up-regulated shoot DEGs associated with GO:0036211 also includes several genes that encode kinases associated with the mitogen-activated kinase cascades, which have been reported to contribute to multiple signaling pathways, including those responding to abiotic and biotic stressors as well as hormonal signaling and developmental regulation [66,67,68].

### 3.6. Genes Associated with the “Translation” GO Group (GO:0006412) Are Down-Regulated in Gaz8 Shoots and Roots under the Microgravity Environment on ISS

As discussed in the previous sections of this manuscript, the three accessions of *Brachypodium distachyon* we tested showed both common and accession-specific transcriptional responses to microgravity, with the latter group predominating. In Gaz8, we noticed a significant association of down-regulated shoot and root DEGs with GO groups related to translation (GO:0006412; Supplemental Table S14), which was not observed in either Bd21 or Bd21-3 accessions. A majority of these genes encode proteins associated with ribosome biogenesis. Previous studies in rice and Arabidopsis have suggested ribosome biogenesis as an index of cell growth and proliferation in cell cultures [69,70]. Whether this microgravity-associated decrease in expression of ribosome biogenesis-related genes can explain the short-root phenotype displayed by Gaz8 seedlings grown on ISS remains unknown. Unfortunately, our experimental set-up did not allow quantification of root tip cell size and numbers of meristematic cells in the root apical meristem.

### 3.7. Root Hair Growth Is Enhanced under Microgravity Conditions

As discussed in the first section of results, seedlings grown under microgravity environment carried longer root hairs compared to ground controls. In an attempt to better understand the molecular mechanisms that govern this phenotype, we investigated the possibility that some root DEGs might be candidate root-hair growth regulators. Unfortunately, there is no published root-hair specific transcription profile in Brachypodium. Therefore, we identified both Arabidopsis and rice orthologs to root microgravity-related DEGs and then verified if any of the orthologs to these root-specific DEGs are also differentially expressed in Arabidopsis and/or rice root hairs [14,71,72].

Starting with rice, this analysis revealed the existence of 19 Bd21 DEGs whose rice orthologs are preferentially expressed in rice root hairs [72]. Unfortunately, only one of these candidate DEGs was up-regulated in Bd21 under microgravity (*Bradi3g20045*), the remaining 18 being down-regulated (Supplemental Table S15). We then investigated whether Arabidopsis orthologs to the Brachypodium root DEGs are also differentially expressed in Arabidopsis root hairs using the microarray data set published in Lan et al., 2013 [71]. Eighteen of the Bd21 root DEGs identified in this study have Arabidopsis orthologs that are also differentially expressed in Arabidopsis root-hair cells with similar expression trends (Supplemental Table S16). Similar comparisons involving Bd21-3 and Gaz-8 accessions identified 11 and 138 DEGs, respectively, with Arabidopsis orthologs showing similar patterns of differential expression in root hair cells [71]. None of these root-hair associated DEGs were identified as differentially expressed in all three accessions. However, Brachypodium orthologs to the Arabidopsis *PIN2* gene (*At5g57090*), which encodes an auxin efflux carrier family protein recently shown to play an important role in root-hair development [73,74,75], were shown to be down-regulated in Brachypodium in response to the microgravity environment (*Bradi2g52640* for Bd21 and Bd21-3 and *Bradi1g31530* for Gaz8; Supplemental Table S16). Unfortunately, there is to date no evidence that a knock-down mutation of *PIN2* will cause a longer root-hair phenotype. Therefore, we currently have no clear molecular explanation for the long root-hair phenotype displayed by Brachypodium seedlings exposed to the microgravity environment of ISS.

An opposite root-hair response to microgravity has been reported for Arabidopsis seedlings (shorter root hairs relative to ground controls) [14]. It should however be cautioned that root-hair growth and development are highly sensitive to a variety of environmental and hormonal constraints such as water, nutrients and oxygen availability, mechanical perturbations, pH and ROS status, exposure to ethylene, etc. [76,77,78]. Importantly, the hardware and experimental designs used in the Arabidopsis study discussed in reference [14] and our Brachypodium experiment differ significantly in several respects. For instance, our Brachypodium seedlings were grown in foam-based APEX Growth Units with constant light exposure, while Kwon et al. grew their Arabidopsis seedlings in agar-based media inside the Biological Research in Canisters (BRIC) hardware under complete darkness. The differences in seedling growth conditions between these two experiments could explain, at least partly, the differences in root-hair responses to microgravity.

## 4. Discussion

In this paper, we describe the morphological and transcriptomic changes displayed by the seedlings of three *Brachypodium distachyon* accessions in response to the microgravity conditions of ISS relative to ground controls. Microgravity-exposed seedlings of all three accessions displayed shorter roots than ground controls, a phenotype that was previously reported for other plant species such as Cress [79], wheat [80], and Arabidopsis [16]. It is however important to note that a decrease in primary root length in response to microgravity is not a general plant response, as other reports have indicated increases in root length for microgravity-exposed seedlings in flax, rice, and soybean [29,81,82,83,84]. The reasons for such variations in root growth responses to microgravity are not known, although differences in genotypes and growth conditions may be responsible. Some of the gene-expression responses to the microgravity environments uncovered in this study may provide some clues, though. Indeed, genes annotated as regulating cell wall extensibility such as xyloglucan endotransglucosylase/hydrolase (*Bradi1g27867*) and expansins (*Bradi1g74720, Bradi3g50740, Bradi3g50730,* and *Bradi3g27440*), were down-regulated in Bd21 Brachypodium roots under microgravity conditions (Supplemental Table S2). Interestingly, knockout mutations in Arabidopsis orthologs to these genes have been reported to restrict cell wall extensibility and limit cell expansion and root elongation [85,86]. Similarly, we observed a tendency for many ribosomal-related genes to decrease in expression in Brachypodium roots under microgravity conditions relative to ground controls in Gaz8 accession (Supplemental Table S13). Previous studies using Arabidopsis cell cultures demonstrated a correlation between ribosomal biogenesis and cell growth, observing a depletion in both processes under both microgravity conditions or simulated microgravity [69,70]. Unfortunately, our experiments were not set up to allow observation of cell numbers in the root apical meristem, or cell elongation in the root elongation zone. Therefore, we cannot determine whether the short-root phenotype displayed by Brachypodium seedlings exposed to microgravity derive from decreased cell proliferation in the root apical meristem or altered cell elongation in the elongation zone.

Our transcriptomic analysis of Brachypodium responses to the microgravity environment on the ISS differentiated between shoot and root responses. This allowed us to demonstrate dramatic differences in expression responses between shoots and roots for all three Brachypodium accessions analyzed. In fact, only 3% of the DEGs identified in this study were similarly differentially expressed between root and shoot samples. Furthermore, GO enrichment analysis of these DEGs uncovered distinct major GO groups as enriched in shoot and root DEGs, respectively. The few GO groups that were similarly over-represented between shoot and root DEGs included genes that showed opposite expression responses to the microgravity environment between organs, as nicely documented by the expression differences between shoots and roots for distinct subgroups of peroxidase genes belonging to the Oxidative-Reduction Process GO group in Bd21 (Supplemental Figure S6). These results are compatible with those reported for *Arabidopsis thaliana* seedlings exposed to microgravity [6]. They suggest that the roots and shoots of young Brachypodium or Arabidopsis seedlings develop distinct response strategies to cope with the constraints dictated by exposure to the microgravity environment on ISS. Alternatively, it is also possible that the roots and shoots of young seedlings exposed to microgravity are subject to different environmental constraints imposed by their distinct functional contributions to plant development.

Similar models can be proposed to explain the vastly distinct transcriptional responses to microgravity displayed by the three Brachypodium accessions analyzed in this study. These accessions evolved under distinct conditions in different ecosystems within the Mediterranean area [87]. The varying selective pressures they encountered during their evolutionary history led to distinct genotypes that likely favored their adaptation to their native environment. When transferred to a new environment such as the microgravity conditions on the ISS, plants belonging to distinct accessions are differentially equipped to respond to these new constraints, leading to diverging root and shoot expression profiles along with differential growth and/or morphological responses. Larger-scale investigations of root and shoot growth and expression responses to microgravity of multiple Brachypodium accessions using genome-wide association studies should, in the longer term, provide exciting new information on key loci contributing to this variation in morphological and molecular adaptive responses to microgravity.

Plants have been identified as key components of bioregenerative life-support systems for long-term space-exploration missions. This is in large part due to their ability to photosynthesize, thereby recycling CO_2_ and wastewater to produce O_2_, carbohydrates, and other biological molecules necessary for plant growth and development. The resulting biomass will constitute a significant source of food, feed, and fiber for the crew. Our data indicate that many photosynthesis-related genes are down-regulated in Brachypodium seedling shoots under microgravity conditions. We showed that such transcriptional responses of photosynthesis-related genes to the microgravity environment of ISS may be associated with oxidative stress exposure leading to an activation of the GUS retrograde signaling pathway and/or activation of *PIF* gene expression. It is interesting to note that hypergravity exposure, which leads to a strengthening of the cell wall, has recently been associated with increased chloroplast size, photosynthesis, and plant growth in moss, possibly reflecting a source–sink link between photosynthesis and carbohydrate utilization in cell wall restructuring [88]. Furthermore, Arabidopsis PIF proteins have been shown to directly bind to the *LAZY4* promoter, thereby activating its expression and modulating the gravitropic response [89]. Therefore, future work will focus on investigating possible mechanistic connections between gravity and microgravity exposure, retrograde signaling between plastids and nucleus, PIF-mediated signaling, photosynthesis, wall restructuring, and carbohydrate metabolism.

In conclusion, our morphological and transcriptomic study of space-grown Brachypodium seedlings provides new insights into monocot plant responses to spaceflight. Because a 1× *g* centrifugation control was not available on ISS for the VEGGIE growth chamber, the responses to spaceflight observed in this project cannot be unambiguously assigned to microgravity exposure. Indeed, some of these responses may also result from exposure to other spaceflight-associated parameters such as radiation, inefficient airflow, high ethylene levels, and/or other experimental parameters.

Some of the responses to spaceflight displayed by *Brachypodium distachyon* seedlings are similar to those from *Arabidopsis thaliana* seedlings, including oxidative stress response, decreased expression of photosynthesis-related genes, and wall restructuring. Those types of general responses also appear to be common between *Brachypodium distachyon* accessions. We also observed some transcriptional responses to microgravity conditions that are more specific to *Brachypodium distachyon* accessions, suggesting a specificity of response that may be expected from plant populations that have been subjected to distinct evolutionary constraints and are now subjected to a totally new environment (microgravity).

Our studies focused on the early stages of seedling development, and it will be important to evaluate the contribution of the identified responses to spaceflight at later phases of plant development and reproduction. A careful evaluation of the contribution of each response, general or specific, to the microgravity environment and its significance for plant survival, growth, and productivity under microgravity should lead to the design of better cultivars and/or cultivation methodologies to produce crops with improved potential for use as key components of bioregenerative life support systems for long-term space-exploration missions. Furthermore, the existence of distinct molecular responses to the microgravity environment between species, and between accessions within species, suggests the need to expand these types of studies directly to cultivated crop species of potential use during spaceflight.

## Data Availability

The RNAseq sequencing fastq files have been uploaded into the GeneLab Data Repository (http://genelab.nasa.gov/data, accessed on 6 February 2023; GLDS-375).

## Supplementary Materials

The following supporting information can be downloaded at https://www.mdpi.com/article/10.3390/life13030626/s1: Figure S1: Blueprint showing the positions of each APEX Growth Unit within the VEGGIE growth chambers both onboard ISS and on the ground; Figure S2. Gene Ontology (GO) enrichment analysis of down-regulated root DEGs from 5-day old *Brachypodium distachyon* seedlings of the Bd21, Bd21-3, and Gaz8 accessions exposed to microgravity conditions on ISS relative to ground-control conditions; Figure S3: Gene Ontology (GO) enrichment analysis of up-regulated root DEGs from 5-day old *Brachypodium distachyon* seedlings of the Bd21, Bd21-3, and Gaz8 accessions exposed to microgravity conditions on ISS relative to ground-control conditions; Figure S4: Gene Ontology (GO) enrichment analysis of down-regulated shoot DEGs from 5-day old *Brachypodium distachyon* seedlings of the Bd21, Bd21-3, and Gaz8 accessions exposed to microgravity conditions on ISS relative to ground-control conditions; Figure S5: Gene Ontology (GO) enrichment analysis of up-regulated shoot DEGs from 5-day old *Brachypodium distachyon* seedlings of the Bd21, Bd21-3, and Gaz8 accessions exposed to microgravity conditions on ISS relative to ground-control conditions; Figure S6: Arabidopsis PIF1 regulatory network connecting transcription factors (purple circles) and target orthologs to Brachypodium Bd21 DEGs associated with the Photosynthesis GO group; Figure S7: The microgravity environment on ISS is associated with increased transcription of phytochrome A (PhyA) and phytochrome interacting factor (PIF) genes (yellow bars) along with decreased expression of photosynthesis-related genes (blue bars) in *Brachypodium distachyon* seedlings; Table S1: RNAseq reads alignment summary; Table S2: List of Bd21 DEGs organized based on organ type (shoot and root); Table S3: List of Bd21-3 DEGs organized based on organ type (shoot and root); Table S4: List of Gaz8 DEGs organized based on organ type (shoot and root); Table S5: List of common DEGs between all three accessions, organized based on organ type (shoot and root); Table S6: List of all Bd21 DEGs belonging to the Oxidation-Reduction Process GO group (GO:0055114) grouped based on tissue type (root/shoot) and mode of regulation (up/down under microgravity relative to ground control); Table S7: List of Arabidopsis genes whose Brachypodium orthologs are differentially expressed in Bd21 roots under microgravity conditions; Table S8: List of Arabidopsis genes whose Brachypodium orthologs are differentially expressed in Bd21 shoots under microgravity conditions relative to ground controls; Table S9: Chi-squared analysis of shoot DEG ortholog association with ROS wheel groups; Table S10: List of 29 transcription factors predicted to regulate the expression of Bd21 shoot DEGs associated with the Photosynthesis GO group under microgravity conditions; Table S11: Bd21 shoot DEGs whose Arabidopsis orthologs are regulated by either the trio of phytochrome-interacting bHLH factors AtPIF1, 4, and 5 (PIF-trio) and/or by AtPIF3 (PIF3; Zhang et al. 2013); Table S12: List of up-regulated shoot DEGs associated with the Regulation of Transcription, DNA-Templated (GO:0006355) Ontology group in Bd21 and Gaz8 accessions; Table S13: List of up-regulated DEGs belonging to the Protein Modification (GO:0036211) Gene Ontology group in Bd21 and Gaz8; Table S14: List of Gaz8 root and shoot DEGs associated with the Translation Gene Ontology (GO) group (GO:0006412); Table S15: List of root DEGs whose rice orthologs display preferential expression in rice root-hair cells (Moon et al., 2018); Table S16: Root DEGs whose Arabidopsis orthologs are also preferentially expressed in Arabidopsis root hairs (Lan et al., 2013).
