# Supplementary material for: Brachypodium distachyon Seedlings Display Accession-Specific Morphological and Transcriptomic Responses to the Microgravity Environment of the International Space Station
Source: Life (Basel). 2023 Feb 23;13(3):626. doi: 10.3390/life13030626 (PMC10058394; doi:10.3390/life13030626)
Supplement: Supplementary file 1 [file life-13-00626-s001.zip › life-2159103-supplementary/Supplemental Figures.pdf]

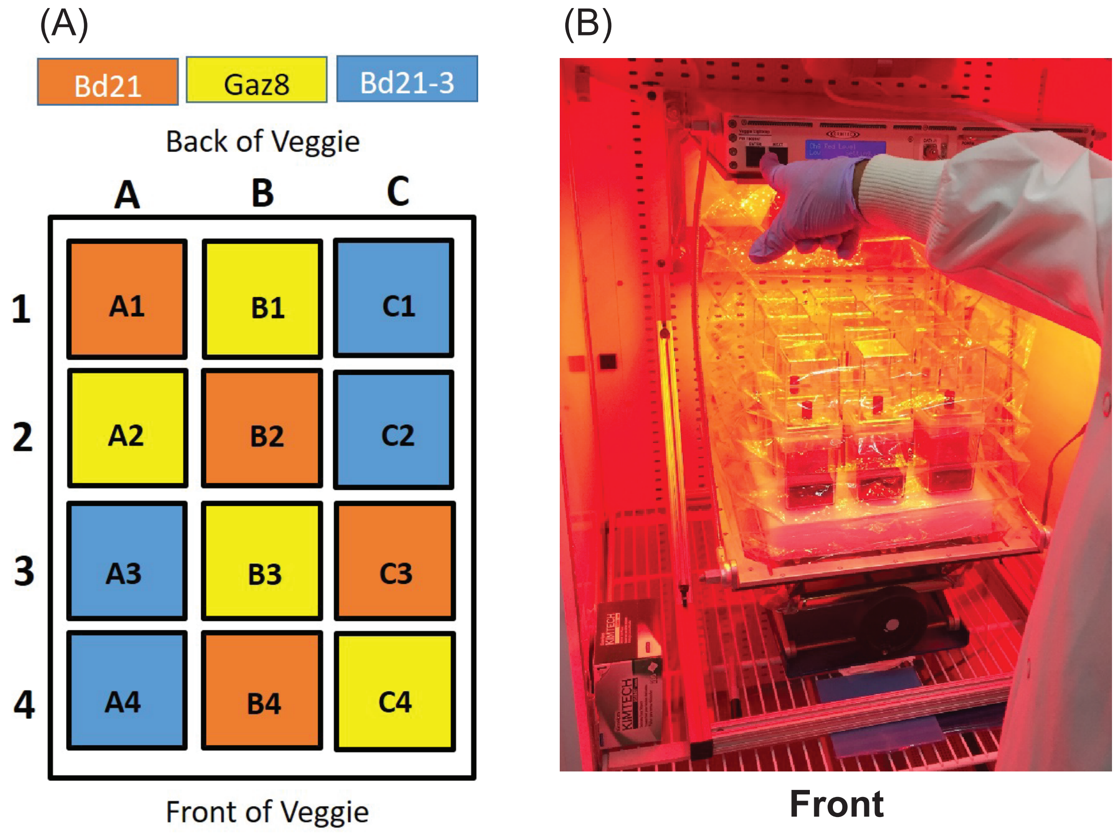

Supplemental Figure S1.

GO_acc	Term	Bd21 FDR	Bd21 Total	Bd21 Query	Bd21-3 FDR	Bd21-3 Total	Bd21-3 Query	Gaz8 FDR	Gaz8 Total	Gaz8 Query
GO:0055114	oxidation-reduction process	5.6E-38	1%	26%	2.9E-06	1%	22%	1.2E-13	1%	22%
GO:0008152	metabolic process	1.4E-04	35%	54%				1.8E-21	35%	70%
GO:0006468	protein phosphorylation	9.1E-03	1%	6%				4.1E-02	1%	4%
GO:0006979	response to oxidative stress	5.4E-09	1%	10%				3.7E-04	1%	5%
GO:0006950	response to stress	2.0E-02	5%	12%						
GO:0044710	single-organism metabolic process	7.4E-10	11%	31%						
GO:0006518	peptide metabolic process							5.7E-61	3%	39%
GO:0043043	peptide biosynthetic process							4.9E-66	2%	39%
GO:0044267	cellular protein metabolic process							8.0E-39	8%	44%
GO:0006807	nitrogen compound metabolic process							1.0E-17	19%	47%
GO:0009058	biosynthetic process							6.1E-18	17%	45%
GO:0043603	cellular amide metabolic process							4.7E-60	3%	39%
GO:0044238	primary metabolic process							2.1E-12	29%	54%
GO:1901566	organonitrogen compound biosynthetic process							2.2E-49	4%	40%
GO:0044237	cellular metabolic process							1.2E-10	30%	53%
GO:1901576	organic substance biosynthetic process							2.1E-19	16%	45%
GO:0044249	cellular biosynthetic process							1.5E-20	16%	45%
GO:0071704	organic substance metabolic process							4.2E-10	32%	55%
GO:0044271	cellular nitrogen compound biosynthetic process							1.3E-28	12%	44%
GO:0034645	cellular macromolecule biosynthetic process							1.3E-28	12%	44%
GO:0044260	cellular macromolecule metabolic process							2.7E-20	21%	52%
GO:0034641	cellular nitrogen compound metabolic process							8.4E-19	18%	46%
GO:0010467	gene expression							2.8E-27	13%	45%
GO:0043170	macromolecule metabolic process							6.5E-20	23%	54%
GO:0043604	amide biosynthetic process							1.2E-65	3%	39%
GO:0019538	protein metabolic process							7.3E-39	9%	46%
GO:0009059	macromolecule biosynthetic process							4.8E-28	12%	44%
GO:0006412	translation							4.9E-66	2%	39%
GO:0009987	cellular process							4.3E-06	38%	56%
GO:1901564	organonitrogen compound metabolic process							3.6E-41	6%	41%

Supplemental Figure S2.

		Bd21			Bd21-3			Gaz8		
GO_acc	Term	*FDR*			*FDR*			*FDR*		
GO:0008152	metabolic process	2.3E-02	Total<br>Query	35%<br>56%	3.4E-05	Total<br>Query	35%<br>59%	5.7E-03	Total<br>Query	35%<br>56%
GO:0055114	oxidation-reduction process	8.8E-03	Total<br>Query	1%<br>9%	6.7E-12	Total<br>Query	1%<br>17%	8.7E-11	Total<br>Query	1%<br>17%
GO:0016310	phosphorylation	1.5E-05	Total<br>Query	2%<br>17%						
GO:0006468	protein phosphorylation	1.5E-02	Total<br>Query	1%<br>17%						
GO:0006793	phosphorus metabolic process	2.9E-03	Total<br>Query	4%<br>17%						
GO:0006464	cellular protein modification process	1.5E-02	Total<br>Query	5%<br>17%						
GO:0043412	macromolecule modification	2.8E-02	Total<br>Query	6%<br>17%						
GO:0036211	protein modification process	1.5E-02	Total<br>Query	5%<br>17%						
GO:0006796	phosphate-containing compound metabolic process	2.9E-03	Total<br>Query	4%<br>17%						
GO:0044710	single-organism metabolic process							7.8E-04	Total<br>Query	11%<br>28%

Supplemental Figure S3.

GO_acc	Term	Bd21			Bd21-3			Gaz8		
		FDR	Total	Query	*FDR*	Total	Query	*FDR*	Total	Query
GO:0016310	phosphorylation	3.8E-03	2%	5%	5.0E-07	2%	20%	8.4E-25	2%	8%
GO:0006796	phosphate-containing compound metabolic process	4.8E-03	4%	8%	2.4E-05	4%	22%	6.7E-18	4%	11%
GO:0008152	metabolic process	3.8E-25	35%	63%	2.4E-06	35%	73%	3.9E-44	35%	59%
GO:0055114	oxidation-reduction process	2.2E-40	1%	15%	4.7E-04	1%	12%	5.9E-54	1%	11%
GO:0006793	phosphorus metabolic process	1.5E-03	4%	8%	2.4E-05	4%	22%	8.8E-18	4%	11%
GO:0044710	single-organism metabolic process	1.2E-12	11%	25%	4.6E-03	11%	29%	1.5E-11	11%	19%
GO:0009116	nucleoside metabolic process	2.3E-02	1%	3%				1.7E-02	1%	2%
GO:0042278	purine nucleoside metabolic process	7.1E-03	1%	3%				2.3E-02	1%	2%
GO:0009167	purine ribonucleoside monophosphate metabolic process	6.8E-03	1%	3%				3.6E-03	1%	2%
GO:0006091	generation of precursor metabolites and energy	1.7E-17	1%	9%				1.1E-10	1%	4%
GO:0006753	nucleoside phosphate metabolic process	1.6E-02	1%	3%				2.1E-02	1%	2%
GO:0019693	ribose phosphate metabolic process	8.6E-03	1%	3%				3.2E-02	1%	2%
GO:0009765	photosynthesis, light harvesting	1.0E-17	0%	4%				1.1E-12	0%	2%
GO:0009144	purine nucleoside triphosphate metabolic process	4.8E-03	1%	3%				1.6E-03	1%	2%
GO:0015979	photosynthesis	2.6E-42	1%	14%				1.3E-34	1%	7%
GO:0046034	ATP metabolic process	3.8E-03	1%	3%				8.7E-04	1%	2%
GO:0009205	purine ribonucleoside triphosphate metabolic process	4.8E-03	1%	3%				1.5E-03	1%	2%
GO:0019684	photosynthesis, light reaction	5.5E-18	0%	6%				2.7E-12	0%	3%
GO:0009141	nucleoside triphosphate metabolic process	8.6E-03	1%	3%				6.3E-03	1%	2%
GO:0009199	ribonucleoside triphosphate metabolic process	7.5E-03	1%	3%				4.6E-03	1%	2%
GO:0006163	purine nucleotide metabolic process	8.2E-03	1%	3%				2.9E-02	1%	2%
GO:0046128	purine ribonucleoside metabolic process	6.8E-03	1%	3%				2.0E-02	1%	2%
GO:0055085	transmembrane transport	1.6E-02	3%	6%				1.4E-04	3%	6%
GO:0009161	ribonucleoside monophosphate metabolic process	1.2E-02	1%	3%				1.2E-02	1%	2%
GO:0009126	purine nucleoside monophosphate metabolic process	6.8E-03	1%	3%				3.6E-03	1%	2%
GO:0009150	purine ribonucleotide metabolic process	6.8E-03	1%	3%				2.1E-02	1%	2%
GO:0009123	nucleoside monophosphate metabolic process	1.3E-02	1%	3%				1.4E-02	1%	2%
GO:0044267	cellular protein metabolic process				1.9E-02	8%	22%	1.7E-11	8%	16%
GO:0071704	organic substance metabolic process				7.6E-03	32%	55%	2.4E-05	32%	40%
GO:0006468	protein phosphorylation				4.5E-08	1%	20%	8.7E-25	1%	7%
GO:0019538	protein metabolic process				3.8E-03	9%	27%	5.6E-20	9%	20%
GO:0006464	cellular protein modification process				1.4E-03	5%	20%	9.7E-04	5%	8%
GO:0043412	macromolecule modification				3.6E-03	6%	20%	9.7E-04	6%	9%
GO:0036211	protein modification process				1.4E-03	5%	20%	9.7E-04	5%	8%
GO:0009259	ribonucleotide metabolic process	1.3E-02	1%	3%						
GO:0009117	nucleotide metabolic process	1.6E-02	1%	3%						
GO:0009119	ribonucleoside metabolic process	1.6E-02	1%	3%						
GO:0072521	purine-containing compound metabolic process	2.2E-02	1%	3%						
GO:0022900	electron transport chain	8.6E-03	0%	2%						
GO:0006518	peptide metabolic process							5.0E-10	3%	8%
GO:1901566	organonitrogen compound biosynthetic process							2.2E-11	4%	10%
GO:0044237	cellular metabolic process							1.5E-07	30%	39%
GO:0006265	DNA topological change							4.3E-03	0%	1%
GO:0015977	carbon fixation							3.1E-03	0%	1%
GO:0043170	macromolecule metabolic process							2.3E-02	23%	27%
GO:0055086	nucleobase-containing small molecule metabolic process							1.2E-02	1%	3%
GO:0006412	translation							3.7E-13	2%	8%
GO:0009987	cellular process							4.5E-04	38%	45%
GO:0009132	nucleoside diphosphate metabolic process							1.1E-02	0%	1%
GO:0043043	peptide biosynthetic process							4.0E-13	2%	8%
GO:0043603	cellular amide metabolic process							1.9E-09	3%	8%
GO:0044238	primary metabolic process							5.2E-07	29%	38%
GO:0006811	ion transport							2.9E-02	2%	3%
GO:0043604	amide biosynthetic process							8.3E-13	3%	8%
GO:1901564	organonitrogen compound metabolic process							1.7E-11	6%	13%

Supplemental Figure S4

GO_acc	Term	Bd21 FDR	Bd21 Total	Bd21 Query	Bd21-3 FDR	Bd21-3 Total	Bd21-3 Query	Gaz8 FDR	Gaz8 Total	Gaz8 Query
GO:0016310	phosphorylation	4.7E-13	2%	13%	5.9E-07	2%	12%	4.7E-13	2%	13%
GO:0055114	oxidation-reduction process	2.6E-14	1%	12%	8.2E-13	1%	15%	2.6E-14	1%	12%
GO:0006796	phosphate-containing compound metabolic process	1.4E-07	4%	14%	2.5E-03	4%	12%	1.4E-07	4%	14%
GO:0008152	metabolic process	9.3E-07	35%	55%	8.1E-09	35%	64%	9.3E-07	35%	55%
GO:0006468	protein phosphorylation	7.7E-16	1%	13%	1.0E-08	1%	12%	7.7E-16	1%	13%
GO:0006793	phosphorus metabolic process	1.4E-07	4%	14%	2.5E-03	4%	12%	1.4E-07	4%	14%
GO:0006464	cellular protein modification process	1.0E-06	5%	15%	2.5E-03	5%	14%	1.0E-06	5%	15%
GO:0036211	protein modification process	1.0E-06	5%	15%	2.5E-03	5%	14%	1.0E-06	5%	15%
GO:0043412	macromolecule modification	4.8E-06	6%	16%	9.0E-03	6%	14%	4.8E-06	6%	16%
GO:0006355	regulation of transcription, DNA-templated	2.9E-02	5%	11%				2.9E-02	5%	11%
GO:0031326	regulation of cellular biosynthetic process	4.6E-02	6%	11%				4.6E-02	6%	11%
GO:0019219	regulation of nucleobase-containing compound metabolic process	3.9E-02	6%	11%				3.9E-02	6%	11%
GO:0044267	cellular protein metabolic process	1.7E-02	8%	16%				1.7E-02	8%	16%
GO:0010556	regulation of macromolecule biosynthetic process	4.6E-02	6%	11%				4.6E-02	6%	11%
GO:0051252	regulation of RNA metabolic process	3.1E-02	6%	11%				3.1E-02	6%	11%
GO:1903506	regulation of nucleic acid-templated transcription	2.9E-02	6%	11%				2.9E-02	6%	11%
GO:2001141	regulation of RNA biosynthetic process	2.9E-02	6%	11%				2.9E-02	6%	11%
GO:0009889	regulation of biosynthetic process	4.6E-02	6%	11%				4.6E-02	6%	11%
GO:0019538	protein metabolic process	3.9E-03	9%	18%				3.9E-03	9%	18%
GO:2000112	regulation of cellular macromolecule biosynthetic process	4.3E-02	6%	11%				4.3E-02	6%	11%
GO:0071704	organic substance metabolic process				4.9E-02	32%	45%			
GO:0044710	single-organism metabolic process				3.1E-06	11%	28%			

Supplemental Figure S5

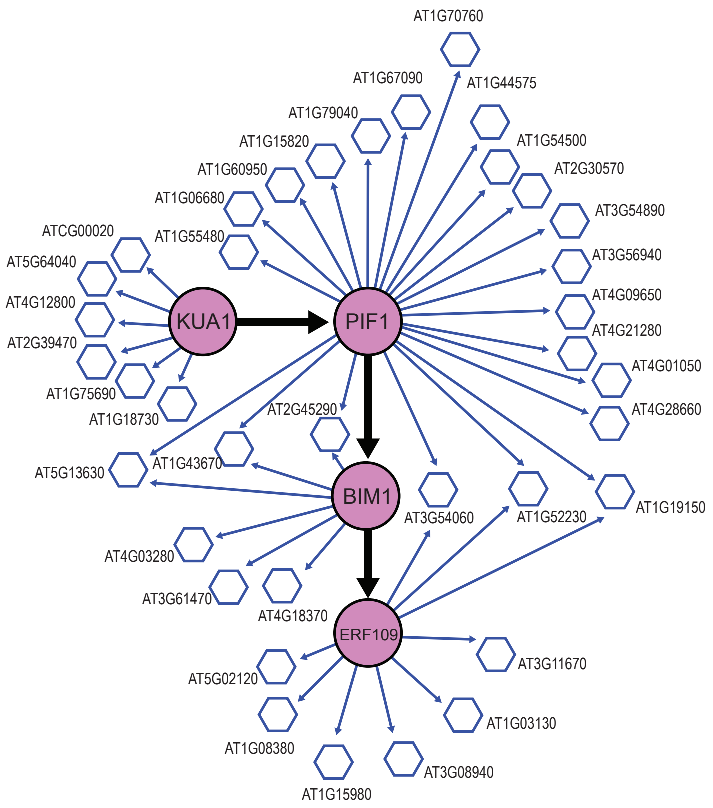

Supplemental figure S6.

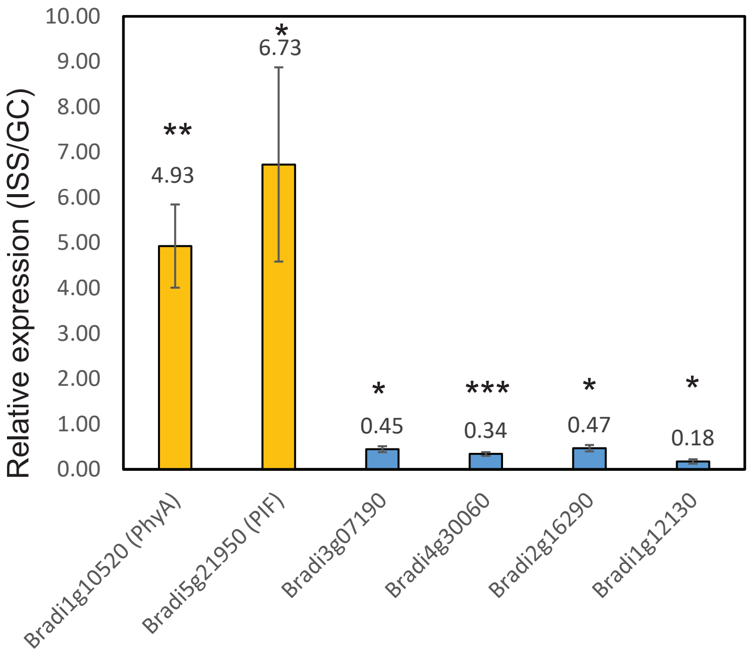

Supplemental figure S7
